# Long Distance Runners Present Upregulated Sweating Responses than Sedentary Counterparts

**DOI:** 10.1371/journal.pone.0093976

**Published:** 2014-04-07

**Authors:** Jeong-Beom Lee, Tae-Wook Kim, Young-Ki Min, Hun-Mo Yang

**Affiliations:** 1 Department of Physiology, College of Medicine, Soonchunhyang University, Cheonan, Chungcheongnamdo, Republic of Korea; 2 Department of Health Care, Graduate School, Soonchunhyang University, Asan, Chungcheongnamdo, Republic of Korea; Universidad Europea de Madrid, Spain

## Abstract

Relatively few studies have investigated peripheral sweating mechanisms of long-distance runners. The aim of this study was to compare peripheral sweating mechanisms in male long-distance runners, and sedentary counterparts. Thirty six subjects, including 20 sedentary controls and 16 long-distance runners (with 7–12 years of athletic training, average 9.2±2.1 years) were observed. Quantitative sudomotor axon reflex testing (QSART) with iontophoresis (2 mA for 5 min) and 10% acetylcholine (ACh) were performed to determine axon reflex-mediated and directly activated (DIR, muscarinic receptor) sweating. Sweat onset time, sweat rate, number of activated sweat glands, sweat output per gland and skin temperature were measured at rest while maximum oxygen uptake (VO_2_max) were measured during maximal cycling. Sweat rate, activated sweat glands, sweat output per gland, skin temperature and VO_2_max were significantly higher in the trained runners than in the sedentary controls. Sweat onset time was significantly shorter for the runners. In the group of long-distance runners, significant correlations were found between VO_2_max and sweat onset time (r^2^ = 0.543, *P*<0.01, n = 16), DIR sweat rate (r^2^ = 0.584, *P*<0.001, n = 16), sweat output per gland (r^2^ = 0.539, *P*<0.01, n = 16). There was no correlation between VO_2_max and activated sweat glands. These findings suggest that habitual long-distance running results in upregulation of the peripheral sweating mechanisms in humans. Additional research is needed to determine the molecular mechanism underlying these changes. These findings complement the existing sweating data in long-distance runners.

## Introduction

Human temperature is regulated within a very narrow range. When exposed to hyperthermic conditions, heat dissipation becomes vital for survival. During exercise, the primary mechanism of heat dissipation is evaporative heat loss secondary to sweat secretion from eccrine glands, particularly when ambient temperature is higher than skin temperature [1]. Studies have shown that physically trained individuals have enhanced capacity for sweat production [2]–[9], which provides a certain physiological advantage when physical exercise is performed under extremely hot conditions [10], [11]. This enhanced capacity for sweat production is achieved via alterations to peripheral sweating mechanisms [8], [9], which include increased gland size, cholinergic sensitivity, sweat glandular output and distribution of gland activity [9].

Different exercise regimens produce different peripheral changes [7]–[9], [12], [13]. For example, eight subjects underwent an short-term physical training (cycle ergometer, 75% VO_2_max, 1 h/day for 10 days, four 15 min exercise periods separated by 5 min rest periods, 25°C) and a heat-acclimation program (cycle ergometer, 50% VO_2_max, 1 h/day for 10 days, two 30 min exercise periods separated by one 15 min rest period, 35°C) [8]. Short-term physical training shifted the vasodilation and sweating thresholds toward lower internal temperatures, and acclimation further lowered these thresholds [8]. In a study of seven long-distance runners (≥42 km), sweating threshold decreased significantly in runners when compared with physically untrained controls either in terms of mean body temperature or esophageal temperature [12]. The runners thus behaved as if the “set point” of their thermoregulatory system had been reset to a lower level [12].

Since the rate of rise in body temperature is one of the important limiting factors to endurance performance [14], physically trained individuals can exercise for longer periods before exhaustion [12], [14]. Of equal importance is the finding that different types of exercise produce different peripheral changes in thermoregulation. For example, a study by Buono and Sjoholm [3] reported that long-distance runners (VO_2_max; male, 65.7±7.1 ml⋅kg^−1^⋅min^−1^, female, 53.4±4.4 ml⋅kg^−1^⋅min^−1^) had more than twice the peripheral sweat rate of their sedentary counterparts (VO_2_max; male, 43.8±6.1 ml⋅kg^−1^⋅min^−1^, female, 37.4±3.7 ml⋅kg^−1^⋅min^−1^) (peripheral sweat rate was determined using pilocarpine iontophoresis). Similary, Irion [13] showed that highly trained long-distance runners (VO_2_max; 62.1±2.3 ml⋅kg^−1^⋅min^−1^) have lower heart rates, higher sweat rates, and lower skin temperatures when compared with track sprinters (VO_2_max; 45.7±0.8 ml⋅kg^−1^⋅min^−1^) at the same work load (during cycling at 30% of VO_2_max for 90 min). Furthermore, sweat rate was significantly correlated with VO_2_max [13].

Henane et al. [7] found that cross-country skiers (50–100 km ski race daily during the winter, long-distance cross-country or bicycle races in the summer) exhibit a higher level of heat tolerance and are better heat-acclimatized compared with swimmers (exclusively of 4 h swimming bouts daily) under a given thermal stress (tympanic temperature clamped at target level of 38°C during 1 h). However, a marked difference in sweating output is observed between skiers and swimmers, despite similar VO_2_max (skiers vs. swimmers; 66.5 vs. 65.8 ml⋅kg^−1^⋅min^−1^).

Distance runners can lose large amounts of body fluid under certain conditions. The primary adaptive change in distance runners may be improved sweating and evaporative efficiency, leading to a reduction in body heat storage and physiological strain. Due to their improved thermoregulatory responses, physically trained individuals are more capable of withstanding higher intensity exercise in hotter conditions than their untrained counterparts.

However, according to Midgley et al. [15], relatively few training studies have been conducted with trained distance runners. Therefore, the aim of this study was to compare the peripheral sweating responses between male long-distance runners and sedentary counterparts and to evaluate the correlation between sweating responses and VO_2_max.

## Materials and Methods

### 2. 1. Subjects

Following approval of experimental protocols from the research committee at the University of Soonchunhyang and obtaining written informed consent, normotensive volunteers were enrolled in the study. All procedures complied with the 1975 Declaration of Helsinki. The subjects were 36 healthy male volunteers from the University of Soonchunhyang and Korea National Sports University and included 20 sedentary controls (subjects who did not perform regular physical activity for the previous 3 years, self-reported) and 16 5–10 km long-distance runners who had 7–12 years of athletic training (average 9.2±2.1 years, with an average of 60–70 km/week, self-reported). General physical characteristics of the subjects are summarized in **Table 1**. The trained runners had significantly lower percent fat and body mass index and a higher VO_2_max than those of the controls. No differences in age, height, weight, or body surface area were observed between the groups. Each subject returned written informed consent to participate in the study, after being thoroughly acquainted with the purpose and the experimental procedures, as well as any potential risks. Subjects were instructed to refrain from alcohol consumption or smoking 24 hours before the test. The volunteers also refrained from medications during the testing period. All experiments were performed at 2–5 p.m. in an automated climate chamber (24.0±0.5°C, 40±3% relative humidity and ≤1 m/s air velocity).

**Table 1:** General physical characteristics.

Groups	Trained	Control
Age (yrs)	23.3±2.20	21.9±1.03
Height (cm)	173.6±5.52	174.1±2.50
Weight (kg)	67.1±8.24	70.4±4.30
BSA (m^2^)	1.80±0.06	1.84±0.05
% fat	14.7±1.36*	21.3±3.03
BMI	22.7±2.03**	24.5±2.45
VO_2_max (ml⋅kg^−1^⋅min^−1^)	57.38±3.99**	43.05±4.54
Career term (yrs)	9.2±2.1	0

Values are mean ± standard deviation. *P<0.05, **P<0.01; significant difference between groups.

Trained subjects (n = 16 male long-distance runners), Control subjects (n = 20 sedentary males), BSA, body surface area, % fat (impedance method), BMI, body mass index, VO_2_max, maximum oxygen uptake, career term, average physical training period.

### 2. 2. The QSART Capsule

**Figure 1:**
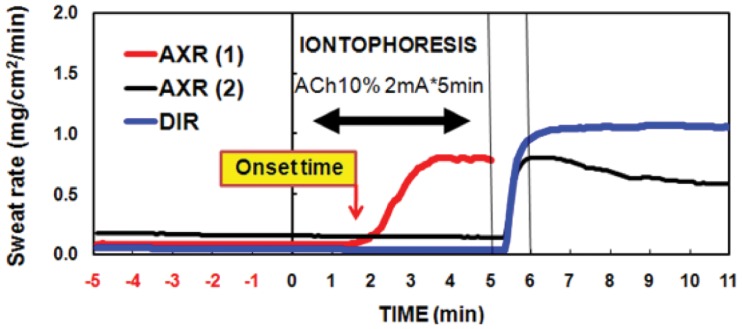
A typical recording during iontophoresis with a 10% acetylcholine solution at 2 mA of direct current applied for 5 min in a single subject. Directly activated and axon-reflex-mediated sweating is shown. Axon reflex-mediated sweating during 0–5 min iontophoresis (AXR1 sweating). Axon reflex-mediated sweating during 6–11 min post-iontophoresis (AXR2 sweating) and directly activated sweating during 6–11 min post-iontophoresis (DIR sweating) are shown.

Recent developments have resulted in the commercial availability of extremely sensitive and reliable sudorometers, which can conveniently analyze and display sweat rates and volumes [16]. The quantitative sudomotor axon reflex test (QSART) is a useful method for evaluating postganglionic sympathetic C fiber function. Iontophoresed acetylcholine (ACh) evokes a measurable and reliable sweat response that has been used to measure autonomic responses [17]. The QSART capsule allows for accurate quantification of directly activated (DIR, muscarinic receptor) and axon reflex-mediated (AXR, nicotinic receptor) sweat responses [18]–[21]. The capsule consists of three concentric compartments. ACh is placed in the outer compartment and directly stimulates the underlying muscarinic receptors of the sweat gland cells (DIR sweating). Additionally, ACh produces axon reflex sweating (**Figure 1**) [20].

### 2. 3. Measurement of Sweat Onset Time and Sweat Rate

Upon arrival at the climate chamber, each subject changed into light clothing and rested quietly for 1 hour. Two QSART capsules were attached to the volar aspect of the left forearm with rubber bands. Capsule 1 was placed midway between the wrist and elbow joints, whereas Capsule 2 was placed 10 cm proximal to Capsule 1. AXR1 data (see **Figure 1**) were obtained from Capsule 1 as partially described by Bickel et al. [22]. The drug solution 10% ACh (Ovisot, Daiichi Pharmaceutical Co., Tokyo Ltd., Japan) was iontophoresed through the skin for 5 min at 2 mA constant current (HV-BIGPAD, Omron, Kyoto, Japan). Immediately following current loading, the capsules were detached and the underlying skin was wiped. The capsules were then swapped. The exchange of capsule locations took ≤20 s for each subject. Data were acquired for another 5 min to permit simultaneous measurement of DIR and AXR2 sweating. ACh-induced sweat output followed a typical time course (**Figure 1**). Sweat output was calculated as the area under the curve 0–5 min AXR1, 6–11 min AXR2, 6–11 min (DIR) and expressed as mg/cm^2^. The time lapse before onset of the AXR1 sweat response was measured as a response latency in minutes (**Figure 1**). Sweat rate was measured according to the capacitance hygrometer-ventilated capsule method [18], [20]. In brief, nitrogen gas was flowed into each compartment at a constant flow rate of 300 ml/min. The change in relative humidity of the effluent gas was detected by a hygrometer (H211, Technol Seven, Yokohama, Japan). Sweating rates were recorded with a PC (PC9801, NEC, Tokyo, Japan) at intervals of 5 s [20], [21]. These measurements were performed while the participants rested quietly in a chair.

### 2. 4. Measurement of Oral and Skin Temperatures and Activated Sweat Gland Density

Oral (sublingual) and skin temperatures immediately adjacent to Capsules 1 and 2 were monitored using thermistors (PXK-67, Technol Seven) connected to a data logger (K-720, Technol Seven) and recorded with a PC (PC9801, NEC) at 5-s intervals [20], [21]. At the end of the QSART recording, the number of activated sweat glands was determined according to the iodine-impregnated paper method [20], [23]. ACh solution was applied to the forearm skin surface near Capsule 2 to directly stimulate the underlying sweat glands and was wiped off. Iodine-starch paper was then pressed against the surface area. The number of blue-black pigment spots in an area of 0.5 cm ×0.5 cm was counted in triplicates under a microscope. The average number of activated sweat glands (number⋅cm^−2^) was calculated. The sweat output per gland (μg⋅min^−1^⋅single gland^−1^) was obtained by dividing the DIR sweating rate (mg⋅cm^−2^ ⋅5min^−1^) by the number of activated sweat glands [20], [21].

### 2. 5. Measurement of VO_2_max

Resting VO_2_ and exercise VO_2_max was measured 2 days after the iontophoresis experiment using an expired air gas analyzer (COSMED; Quark Pulmonary Function Testing Lung Volumes Module 2 ergo, Rome, Italy). Each subject exercised on an electrically braked cycle ergometry (Cateye EC-1000, Tsuyama Manufacturing, Osaka, Japan) to calculate exercise VO_2_max (ml⋅kg^−1^⋅min^−1^). For the evaluation of athletes’ VO_2_max, it is important to select an exercise situation that allows optimal use of the trained muscle fibres [24]. Therefore, cycling was selected as the mode of exercise for this investigation to minimize any advantage distance runners might already have due to the continuous aerobic nature of the work out. Physical load was increased gradually until the subject became exhausted.

### 2. 6. Statistical Analysis

Data are presented as means ± standard deviations. Correlations between VO_2_max and sweat responses were analyzed by Pearson’s correlation coefficient (r). The sweating responses and various temperatures were compared using the paired and independent *t*-test to compare within and between groups. Significant differences were assumed for *P*<0.05.

## Results

### Differences in VO_2_max, Skin and Oral Temperatures and Sweating Parameters

VO_2_max was significantly higher in trained subjects than that in controls (57.38±3.99 vs. 43.05±4.54 ml⋅kg^−1^⋅min^−1^, *P*<0.01), indicating better aerobic capacity in the trained subjects (**Table 1**). Pre- and post-test oral temperatures tended to be lower in the trained subjects than in the controls (pre, 36.53±0.49 vs. 36.63±0.44°C; post, 36.59±0.45 vs. 36.65±0.45°C). However, no significant difference was observed. Pre- and post-test skin temperature was significantly higher in trained subjects than in controls (pre, 32.54±0.46 vs. 32.12±0.39°C, *P*<0.05; post, 33.23±0.49 vs. 32.46±0.40°C, *P*<0.01, respectively) (**Table 2**). Sweat onset time for AXR1 was 0.31 min shorter in trained subjects than in controls (1.34±0.24 vs. 1.65±0.35 min, *P*<0.001). Trained subjects demonstrated higher values of AXR1 (2.94±0.48 vs. 2.01±0.46 mg⋅cm^−2^, *P*<0.001), AXR2 (4.88±0.62 vs. 3.60±0.87 mg⋅cm^−2^, *P*<0.001) and DIR (6.78±0.62 vs. 5.07±0.64 mg⋅cm^−2^, *P*<0.001) than controls (**Table 3**). Activated sweat glands and sweat output per gland were also higher in trained subjects than in the controls (105.5±8.84 vs. 95.46±13.7 counts⋅cm^−2^, *P*<0.05 and 12.91±1.32 vs. 10.76±1.52 μg⋅min^−1^⋅single gland^−1^, *P*<0.001 respectively, **Table 3**).

**Table 2:** Changes in skin temperature and oral temperature in trained and control subjects taken before and after iontophoresis.

	Skin temperature (°C)		Oral temperature (°C)	
Groups	Pre-test	Post-test	Pre-test	Post-test
Trained	32.54±0.46*	33.23±0.49**,^##^	36.53±0.49	36.59±0.45
Control	32.12±0.39	32.46±0.40^#^	36.63±0.44	36.65±0.45

Values are mean ± standard deviation. *P<0.05, **P<0.01; significant difference between groups. ^#^ *P*<0.05, ^##^ *P*<0.01; significant difference between Pre-test and Post-test (pre-iontophoresis vs. post-iontophoresis).

Trained subjects (n = 16 male long-distance runners), Control subjects (n = 20 sedentary males).

**Table 3:** Comparison of sweat onset time for AXR1, sweat rate (AXR1, AXR2 and DIR), activated sweat gland density and activated sweat output per gland.

		Groups	
		Trained	Control
Onset time of AXR1 (min)		1.34±0.24***	1.65±0.35
Sweat rate (mg⋅cm^−2^⋅5min^−1^)	AXR1 0–5 min	2.94±0.48***	2.01±0.46
	AXR2 6–11 min	4.88±0.62***	3.60±0.87
	DIR 6–11 min	6.78±0.62***	5.07±0.64
Activated sweat gland	Density (counts⋅cm^−2^)	105.5±8.84*	95.46±13.7
	Output per gland (μg⋅min^−2^⋅gland^−1^)	12.91±1.32***	10.76±1.52

Values are mean ± standard deviation. *P<0.05, ***P<0.001; significant difference between groups.

DIR, directly activated sweating (muscarinic receptor mediated sweating activity) 6–11 min; AXR, axon reflex-mediated (indirectly activated) sweating (nicotinic receptor mediated), AXR1, measurement from 0–5 min, AXR2, measurement from 6–11 min; Trained (n = 16 male long-distance runners); Control (n = 20 control males).

### Correlation between VO_2_max and Sweat Data

**Figure 2:**
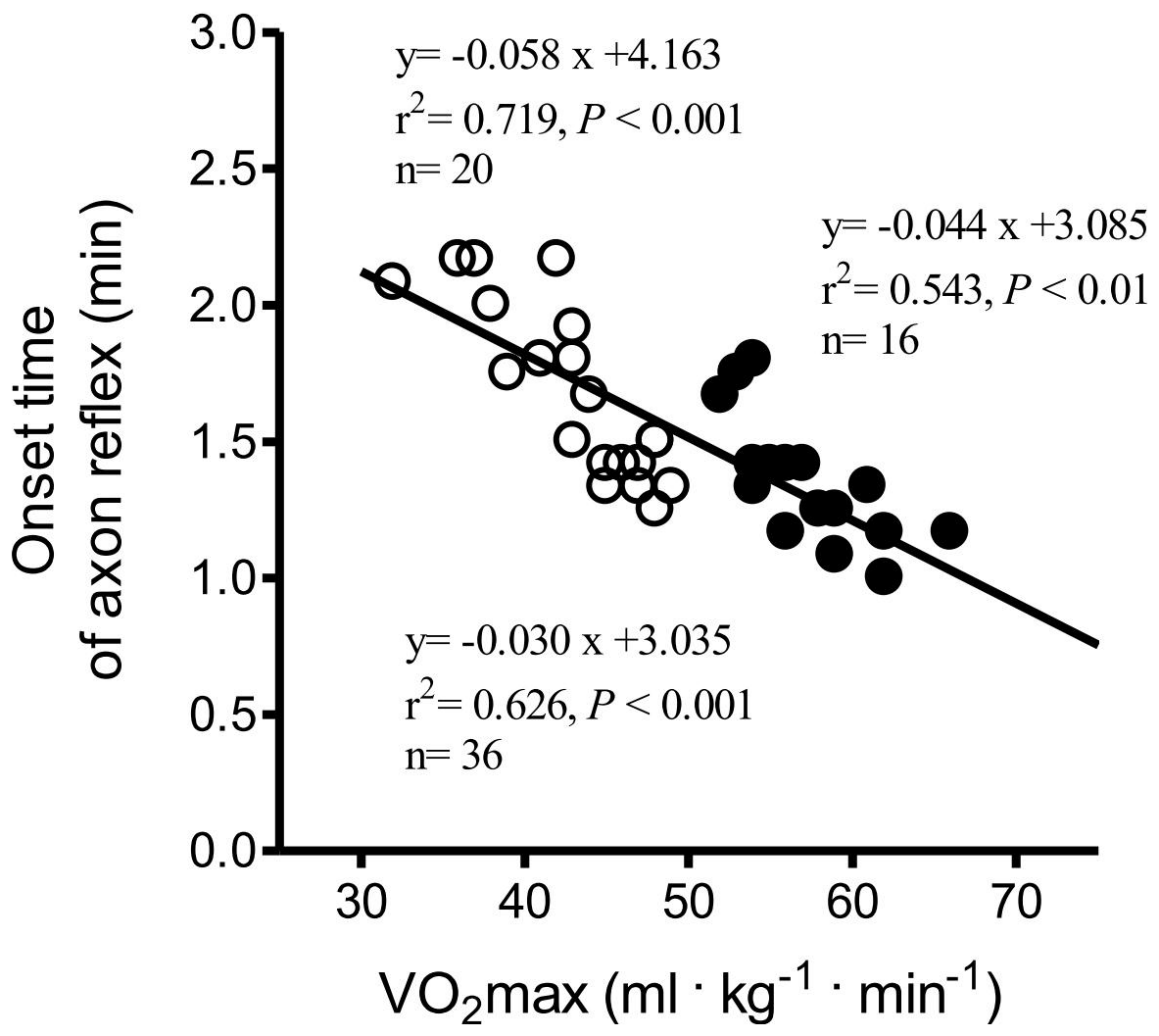
Correlation between VO_2_max and AXR1 sweat onset time in the control subjects (white circles) and trained subjects (black circles). AXR, axon reflex-mediated sweating during measurement 0–5 min.

**Figure 3:**
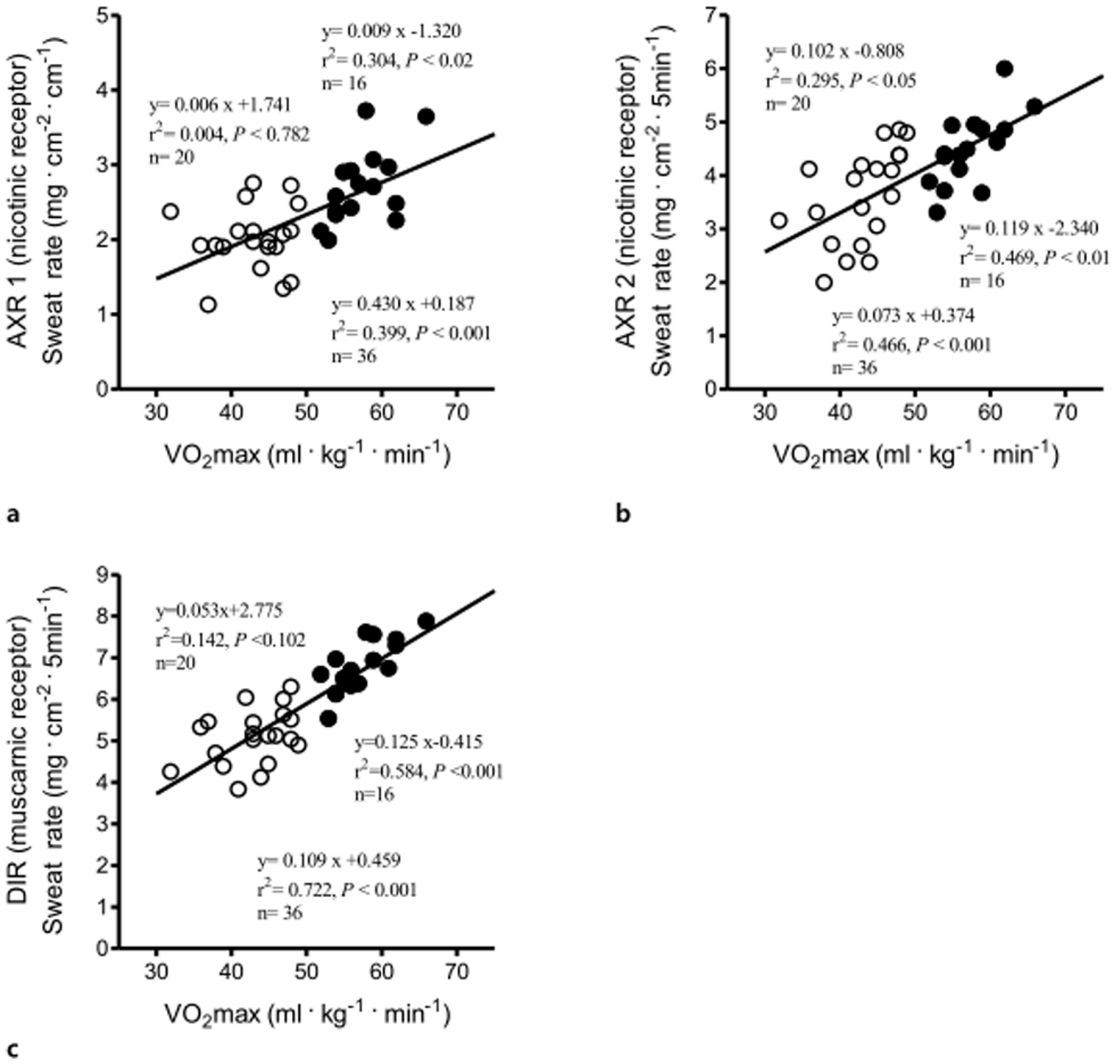
Correlation between VO_2_max and AXR1 (a) AXR2 (b) and DIR (c) sweat rate in control subjects (white circles) and trained subjects (black circles). AXR, axon reflex-mediated sweating. AXR1, measurement during 0–5 min, AXR2, measurement during 6–11 min and DIR, directly activated sweating during measurement 6–11 min.

**Figure 4:**
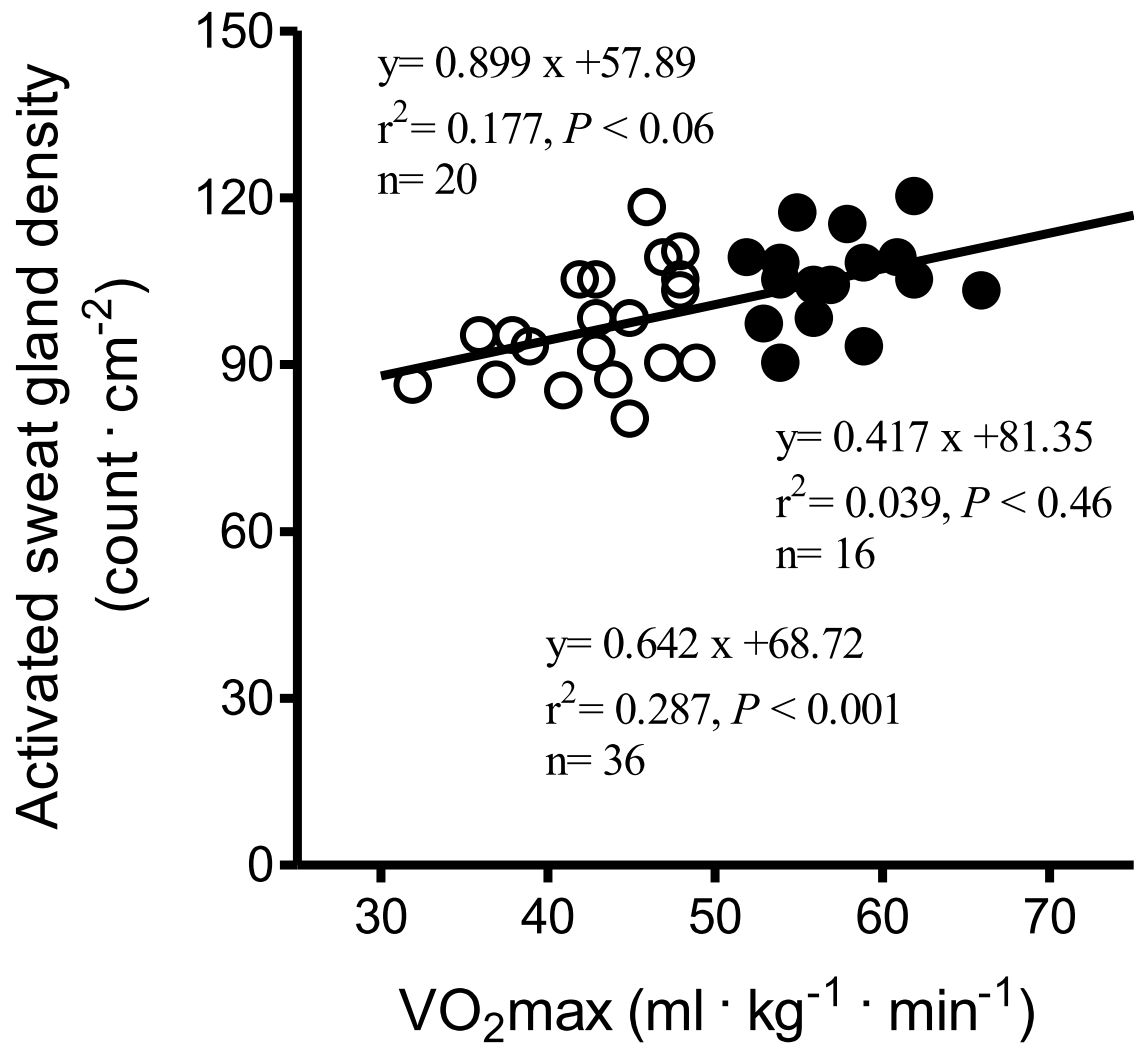
Correlation between VO_2_max and activated sweat glands in control subjects (white circles) and trained subjects (black circles).

**Figure 5:**
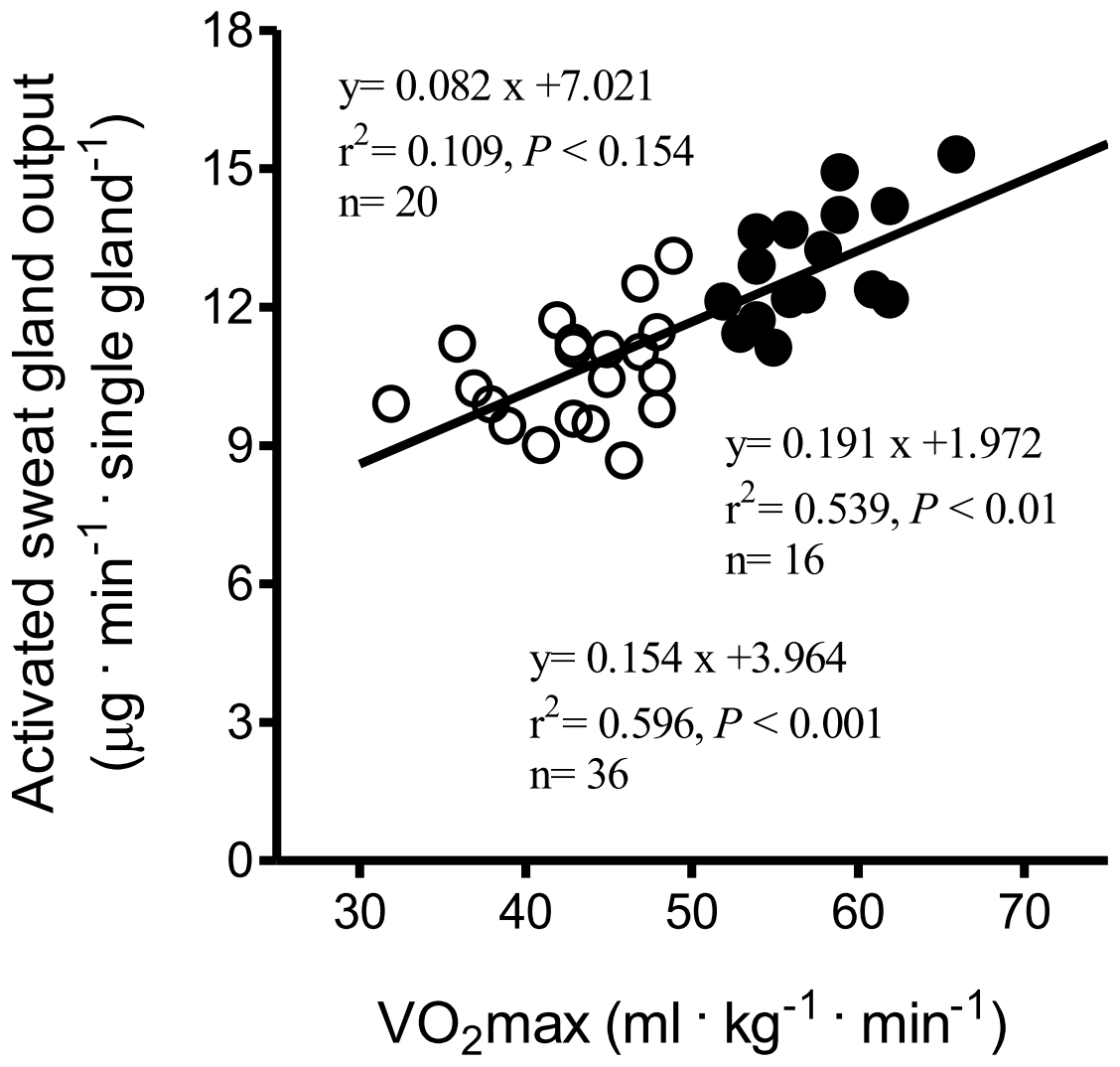
Correlation between VO_2_max and sweat output per gland in control subjects (white circles) and trained subjects (black circles).

In the group of long-distance runners, there were significant correlations between VO_2_max and direct (muscarinic receptor mediated sweating activity) sweat onset time (r^2^ = 0.543, *P*<0.01, n = 16, **Figure 2**), as well as with DIR sweat rate (r^2^ = 0.584, *P*<0.001, n = 16, **Figure 3c**). There was no relationship between VO_2_max and activated sweat glands in the trained subjects (r^2^ = 0.039, *P*<0.46, n = 16, **Figure 4**). However, significant correlations between VO_2_max and sweat output per gland in the group of long-distance runners (r^2^ = 0.539, *P*<0.01, n = 16, **Figure 5**). VO_2_max was not related to activated sweating, sweat onset time, sweat output per gland or activated sweat glands in the controls. Thus, the sweat response of individuals in the control group had a lower correlation with aerobic capacity. The correlation between VO_2_max and either AXR1 (r^2^ = 0.304, *P*<0.02, n = 16, **Figure 3a**) or AXR2 sweat rate (r^2^ = 0.469, *P*<0.01, n = 16, **Figure 3b**) was not significant in the trained subjects.

## Discussion

The main finding of this study was that long-distance runners have greater capacity for peripheral heat dissipation. A significant and strong correlation was found between VO_2_max and DIR sweating in the trained subjects but not in the controls. In addition, trained subjects had a shorter sweat onset time and higher sweat output per gland than those of control subjects for AXR1, AXR2, and the DIR response. We conducted QSART over 11 min to simultaneously examine AXR and DIR. **Figure 3a and 3b** show the significant correlation between VO_2_max and the AXR response induced by iontophoretic ACh, which was greater in the trained subjects than in the controls. Cutaneous application of iontophoretic ACh induces vasodilatation [25]. An increased skin vasodilatory response is associated with increased skin temperature [26], which in turn synchronizes sweat expulsion [27], [28], [29]. Conversely, a decrease skin temperature is associated with decreased sweat rates [30].

Long-term heat acclimation has been shown to shorten sweat onset time, in contrast to short-term heat acclimation, which is not known to induce this change [31]. In the present study, trained subjects had 34–46% higher sweat rates compared to those of controls. The trained subjects had shorter AXR1 sweat onset time. These results are in agreement with the study by Baum et al. [12], in which the sweating threshold of long-distance runners (≥42 km) appeared to have been reset to a lower level. Sweating occurs at a lower core temperature and the slope of the sweat rate to core temperature relationship is higher [2], resulting in a more efficient sweating response.

The trained subjects in our study had significantly higher skin temperature at the site of iontophoresis and slightly lower oral temperature compared to those of controls. However, the significance of these differences are negligible because skin temperature and oral temperature were already at basal levels (32–33°C and 36°C, respectively) **(** **Table 2** **)**.

Different modalities of exercise-induced heat stress elicit various thermoregulatory adaptations. For example, runners can attain higher sweat rates than swimmers in 35°C water [32]. Endurance training leads to peripheral vascular adaptations in skeletal muscles that enhance perfusion and vascular flow capacity. These adaptations may stem from structural modifications of the vasculature and/or alterations in the control of vascular tone. One potential mechanism through which vascular control may be modified is through adaptive changes in the intrinsic responsiveness of vascular endothelium [33].

Regular exercise training in rats increases endothelial function [34] through ACh-induced vasorelaxation, mediated by M3 muscarinic receptors [35]. Acute exercise studies in rats by Cheng et al. [36] showed that receptor-mediated vasodilation responses may be influenced by the quantity of endothelial receptors or by receptor affinity. In our human subject study, routine exercise may be responsible for a similar adaptive (peripheral vascular) phenomenon that improved the sweating response. The trained subjects had a mean exercise VO_2_max that was 33% higher than the controls subjects. VO_2_max is the gold standard of cardiovascular fitness and it usually increases after endurance exercise training. Cardiovascular fitness can significantly influence the degree of sweat loss during exercise [35], [37], [38], in part because of changes in VO_2_max and heat acclimatization. Our findings are in agreement with previous investigations as long-distance runners had higher DIR sweat output (6.78±0.62 mg⋅cm^−2^) compared to controls who had a value of 5.07±0.64 mg⋅cm^−2^ **(** **Table 3** **)**. Moreover, a strong correlation was found between VO_2_max and DIR sweating in the trained subjects (r^2^ = 0.584, *P*<0.001) but this correlation did not exist for controls, who were not as aerobically fit. Buono et al. [5] similarly found that VO_2_max is significantly correlated with maximal sweat rate (r^2^ = 0.76) in distance runners. However, other studies have reported no such correlation [3], [7]. This may be due to differences in exercise type and regimen. For example, Bittel and Buguet [38] found that sweat output increased by 17.3% without any change in VO_2_max for 6 days following endurance training in a cool climate. The strong relationship between VO_2_max and sweating volume further affirms the health benefits of maintaining aerobic fitness. The number of activated sweat glands in the trained subjects was not significantly different from those in the controls, unlike sweat output per gland (*P*<0.001) between the trained and control subjects. Kondo et al. [39] suggested that changes in sweating rate rely on both activated sweat glands and sweat output per gland during the initial period of exercise stage when thermoregulation is passive, whereas further increases in sweating rate are dependent on increases in sweat output per gland. Therefore, the lack of a strong correlation between VO_2_max and activated sweat glands is not surprising.

## Conclusion

In conclusion, routine long-distance runners exhibited higher sweat responses after evoked sweating due to shorter sweat onset time and higher sweat output per sweat gland. Therefore, our findings suggest that routine long-distance running result in upregulated sweating responses in peripheral sweating mechanisms. However, additional research is needed to identify the molecular mechanism underlying these changes. Our results complement existing understanding of perspiration in long-distance runners.
